# Determinants of late antenatal care follow up among pregnant women in Easter zone Tigray, Northern Ethiopia, 2018: unmatched case–control study

**DOI:** 10.1186/s13104-019-4789-8

**Published:** 2019-11-19

**Authors:** Gebrehiwot Gebremariam Weldearegawi, Berhane Fseha Teklehaimanot, Hirut Teame Gebru, Znabu Asfaw Gebrezgi, Kidanemaryam Berhe Tekola, Mulu Ftiwi Baraki

**Affiliations:** 1 Department of Public Health, College of Medicine and Health Sciences, Adigrat University, Adigrat, Ethiopia; 2 School of Public Health, Department of Nutrition, Mekelle University, Mek’ele, Ethiopia; 3 Department of Midwifery, College of Medicine and Health Sciences, Adigrat University, Adigrat, Ethiopia

**Keywords:** Late, Antenatal, Eastern zone, Tigray, Ethiopia

## Abstract

**Objective:**

The purpose of the study was to identify determinants of late antennal care at first visit in health facilities of eastern zone of Tigray, Northern Ethiopia 2018.

**Result:**

Women with unplanned pregnancy (AOR = 4.03, 95%, CI 1.56–5.67), Participants whose previous first antenatal care was after 16 weeks (AOR = 3.9, 95% CI 1.98–7.68), Participants did not accompanied by their partner for antenatal visit (AOR = 1.29, 95%, CI 1.05–4.67), women recognized their current pregnancy at 3 months or late (AOR = 4.7, 95%, CI 2.49–9.04) and participants provided adequate time for their previous antenatal care by health professionals (AOR = 0.461, 95% CI 0.342–0.875) were found the determinant factors of late antenatal care at first Visit. Hence family planning utilization, times of first visit antenatal, information flow and supporting by partners have a great role in improving antenatal care at first visit. There for responsible bodies should give focuses on utilization of family planning, increasing awareness of pregnancy symptoms and health provisional provide adequate time during visits.

## Introduction

Maternal mortality due to complications of child birth in sub Saharan African countries is the highest, which accounts about 66% from the total mortality in the globe [1]. Evidences showed the most common causes of maternal mortalities can be prevented through quality of ANC (antenatal care) [2]. Therefore the World Health Organization recommended all pregnant women should provide the focused ANC services within the first trimester of pregnancy, that enables them with a number of interventions important for themselves and their infants [2, 3]. Similarly of quality ANC is recognized as an important opportunity for screening and early identification of complications such as pre-eclampsia, anaemia, and gestational diabetes [4]. However pregnant women who provided poor quality, attended less and late first visit were associated with an increased risks of stillbirth [5]. Although, the focused ANC model recommends all pregnant woman need to start first visit of ANC in the first trimester of pregnancy, a significant proportion of women from developing countries including Ethiopia did not start ANC according to the recommendation [6, 7].

In the other hand, despite the fact that pregnant women in most of the developing countries attended first ANC at a late time, but it plays a significant role in timely management and treatment of complications to reduces maternal morbidity and mortalities during antepartum, intra partum and post-partum period and a good basis for appropriate management for delivery and after childbirth if they attended early [8]. However, in case of delay attending it resulted with different problems during pregnancy, delivery, and puerperium periods [4, 6]. Though a study done in south Africa, indicated there was no effect of gestational age at first ANC visit on stillbirth outcomes [9], but evidence from New Zealand and south Africa implied late attending of ANC was highly related with still birth [5, 10]. To alleviate the problems related with attending late ANC at first visit, the Federal Ministry Health of Ethiopia is trying to implement the WHO recombination focused ANC model, but many studied in the country indicated only about one-third of pregnant women were attending first ANC before the first 4 months of pregnancy [2, 7, 8, 11]. In addition, even though there are few evidences on the timing of antenatal care in Ethiopia, but the evidences did not addressed the determinants of early antenatal care visit in the country. Hence this study had identified the determinates of late antenatal care among pregnant women who were attending ANC in health facilities of Eastern Zone of Tigray regional state, North Ethiopia 2018.

## Main text

### Methods

#### Study setting and design

The study was done in eastern zone of Tigray regional state, North Ethiopia which was financially funded by Adigrat University with registration number of AGU/CMHS/033/10. Unmatched case–control study design was employed to generalized the determinates of late ANC for all age groups, residences and other related issues from January 2018 to April 2018. Time of first ANC attendance was considered as an outcome variable. Participants were categorized as cases and controls based their time of first ANC as an outcome variable to identify the determinants of late ANC due to the fact that the WHO recommends pregnant women need to start ANC first visit with in the first trimester (in the first 4 months), second visit 20–24 weeks, third visit 28–32 weeks and fourth visit at 36 weeks or after. Accordingly, Participants who visited first ANC after 16 weeks of gestation were considered as cases and participants attended first visit of ANC within the first 16 weeks were considered as controls. All pregnant women who were doing their schedule of first visit in all of the health facilities were included as sources of population. However pregnant women who were severely ill and mentally ill during the data collection were excluded due to they might not give appropriate information.

#### Sampling size and techniques

Sample size was calculated using EPI INFO Version 7 by considering proportion of mothers who had good knowledge on advantage of early ANC among cases was 58.2% (main exposure variable with AOR = 2.1) from previous study done in Ethiopia [7]. In addition 95% CI, 80% power and 1:1 control to case ratio was taken. Accordingly by adding 10% none response rate the final sample size was 201 cases and 201 controls (total of 402 participants). To obtain the required sample size 12 health facilities were selected randomly from the total 44 health facilities of the Zone. Thus, eligible Participants were shared proportionally to population size of each health facilities. Finally participants were selected using systematic sampling technique from the total pregnant mothers attending ANC first visit in the selected health facilities.

#### Data collection tools and analysis

The determinants of late ANC was assessed using structured and pre tested questionnaires by face to face interview. Questionnaires were developed in English, and then it was translated to local language to check its completeness, consistence, accuracy and finally applied the English version. The actual data was collected during their first ANC visit. Twelve Midwifery professional data collectors and three Bachelor of Science in Nursing Supervisors were recruited. Two days training was given for data collectors and supervisors. The preliminary data was coded and checked for completeness, consistent and managed accordingly. Data clean up and cross-checking was also done before the analysis. Data was entered to SPSS version 20 for analysis. Cross tabulation was done to see the distribution of cases and controls by frequency, percentage and mean. Bivariate and multivariate logistic regression was done. Each variable with the outcome of interest at p < 0.05 in the bivariate analysis was transported to multivariable analysis. Each independent variable at p < 0.05 was declared as determinate factors in the final model. Multi-collinearity using VIF (Variance Inflator Factor) at the cut of point 10 and Model goodness of fit using Hosmer–Lemeshow test at *p* value > 0.05 was done. The overall design, data collection and analysis was followed and checked by the funding agency (Adigrat University). Finally the finding of the study was presented to Adigrat University and respected districts.

### Result

#### Part I: Socio demographic characteristics and obstetric history of participants

In the current study, 199 controls and 199 cases were participated making a response rate of 98.7%. Majority, 54 (27.1%) control and 53 (26.6%) case were in the age group of 25–29 years with the mean age of 27 years (SD ± 6.3). The highest 113 (56.8) controls and 109 (54.8) case were house wives. Only 19 (9.5%) controls and 15 (6.5%) case were governmental employed. In educational status, the highest 89 (44.7%) controls and 72 (36.1%) cases were attended 7–10 grade (Table 1).

Most of the participants, 67 (49.6%) controls and 58 (35.4%) cases had 2–4 births. Regarding the number of alive children they had, 69 (53.1%) controls and 77 (47%) cases had 1–2 alive children, and 25 (21.2%) controls and 29 (18.8%) cases had history of abortion. Out of the total participants 53 (26.6%) controls and 84 (42.4%) controls their current pregnancy were unplanned. Concerning the time of previous ANC follow up, 35 (53.3%) controls and 71 (51.1%) case were attended after 16 weeks. Participants were asked if they know when ANC visit will be started, accordingly, 42 (21. 1%) controls and 116 (58.3%) case replied it should start after 16 weeks (Table 2).

#### Part II: Determinates of late ANC among pregnant women in Eastern Tigray, North Ethiopia

The current study identified pregnancy status, Time of previous ANC attendance, accompanied by their partner for ANC, time of recognized their pregnancy and provided adequate time for their previous ANC by health professionals were found the determinates of Late ANC follow up. Participants whose pregnancy was unplanned were 4 times more likely attending late (AOR = 4.03, 95% CI 1.56–5.67). Women whose previous first ANC was after 16 weeks were 3.9 times attended late compared to those whose previous ANC was before 16 weeks (AOR = 3.9, 95% CI 1.98–7.68). Pregnant who recognized their pregnancy after the first 3 months were 4.7 times attending late as compared with those who recognized with in the first 2 months (AOR = 4.75, 95%, CI 1.495–9.042). However participants who provided adequate time for their previous ANC visit by health professionals were about 53% attending early compared to those who did not provided adequate time (AOR = 0.461, 95% CI 0.342–0.875) (Table 3).

The current study revealed that women with unplanned pregnancy were 4 times more likely attending late compared to women their pregnancy was planned. Similar finding was shared from studies in Arbaminch and Addis Ababa, Ethiopia [6, 12]. Bayou et al., also reported intention of pregnancy was found as a predictor of late ANC [13]. Another study in South western Ethiopia explained, late attendance of ANC was higher among women with unplanned pregnancy [14]. An evidenced from South Africa and Kenya indicated, unplanned pregnancy was an independent determinant factor for late ANC [10, 15]. This could be due to pregnant women with unplanned pregnancies might miss supports from partner or family, so they might not recognized their pregnancy early. In contrary if they recognized their pregnancy early, they can alert about the disadvantage attending late and they may give more care for their pregnancy themselves and from spouses.

We found women who attended ANC first after 16 weeks for past pregnancy was showed significant determinant for late attending. Similarly, Girmatsion et al. stated women who attended early ANC for past pregnancy were less likely to start late compared to those attended late for the past pregnancy [7]. This might be the fact that women who attended ANC with in the first 4 months for the past pregnancy are expected to have better awareness on the advantage of early ANC visit. In addition, the odds of late ANC among women who did not accompanied by their partner were 1.2 times higher than those who accompanied. Similar report was observed in a study done in Tanzania [16] and in Ethiopia [12].

Again the odds of late ANC at first visit were 1.2 times higher among women who gave birth 2–4 children than primigravida. Tolefac et al. reported, the odds of late ANC were high among women who had ≥ 4 children [17]. Manzi et al. and Ochako et al. also share similar finding [15, 18]. The same evidence was also shared from a study done in Bhutan [19]. Ideally, as the size of children increases, the likelihood of attending ANC visit early will be dropped. It might be due to in developing countries especially in Ethiopia mothers are responsible and preoccupied in routing house hold activities and giving care for their kids, so they may get difficult in representing another person who gives care for the kids and the house hold activities. This evidence was confirmed by time constraint with household activity was one of the main reason for late ANC in Ethiopia [11]. In the other hand the current study identified women who recognized their pregnancy at third months or late were attending late than those who recognized their pregnancy before 3 months. This finding was supported by a study done in south eastern Tanzania [16].

Hence, the study identified women need to have planned pregnancy, they should recognized their pregnancy early and the health providers should give them adequate time. Tigray regional health bureau and the respective health facilities in collaboration with other stake holders should give due emphasis on community awareness in family planning, sign and symptoms of pregnancy.

## Limitation

The study was case–control study that did not address the outcomes of late attending of ANC first visit, so it will be a focus for future researchable area.

## Data Availability

All data pertaining to this study is attached its description in the annex part at the final document and the data set has been attached in the Journal manuscript tracking system as supporting file coded as “0” for controls and “1” for cases with the file name clean data.

## Appendix

See Tables 1, 2 and 3.

**Table 1:** Socio demographic and economic characteristics of pregnant mothers attending antenatal care follow up Easter Zone Tigray, 2018

S. no	Variables	Characteristics	Before or at 16 weeks (n = 199) controls	After 16 weeks (N = 199) Case	Total
1	Age	< 19 years old	15 (7.5)	18 (9)	33
		20–24 years old	72 (36.2	47 (23.6)	119
		25–29 years old	54 (27.1)	53 (26.6)	107
		30–34 years old	32 (16.1)	55 (27.6)	87
		≥ 35 years old	26 (13.1)	26 (13.1)	52
2	Marital status	Married	176 (88.4)	169 (84.9)	345
		Single	17 (8.5)	20 (10.1)	37
		Divorced	3 (1.5)	10 (4.5)	13
3	Religion	Orthodox	167 (83.9)	163 (81.9)	330
		Muslim	36 (18.1)	33 (16.6)	68
4	Ethnic group	Tigrian	175 (87.9)	179 (89.9)	354
		Amhara	8 (4)	6 (3)	14
		Oromo	6 (3)	7 (3.5)	13
		Others (Afar and SNNP)	6 (3)	7 (3.5)	13
5	Occupational status	Governmental employed	19 (9.5)	15 (6.5)	34
		Student	18 (9)	22 (11.1)	40
		Housewife/farmer	109 (54.8)	113 (56.8)	222
		Merchant	14 (7)	15 (7.5)	19
		Private company	36 (18.1)	29 (14.6)	65
		Others	5 (2.5)	3 (1.5)	8
6	Husband occupation	Governmental employed	33 (16.6)	31 (15.6)	64
		NGO and students	17 (8.5)	9 (4.5)	26
		Farmer	34 (17.1)	41 (20.6)	75
		Merchant	51 (25.6)	49 (24.6)	100
		Private employed	51 (20.7)	53 (26.5)	104
		Daily laborer	13 (6.5)	16 (8.2)	29
7	Educational status	Illiterate	36 (16.1)	54 (27.1)	90
		Can read and write	10 (5)	14 (79)	24
		1–6 grade	33 (16.6)	34 (17.10)	67
		7–10 grade	89 (44.7)	72 (36.1)	161
		Preparatory completed	9 (4.5)	9 (4.5)	18
		Diploma and above	22 (11.1)	16 (8)	38
8	Husband educational level	Illiterate	12 (6)	34 (17.1)	46
		Can read and write	18 (9)	28 (14.1)	46
		1–6 grade	36 (18.1)	25 (12.6)	61
		7–10 grade	76 (38.2%)	71 (35.7)	147
		Preparatory completed	13 (6.5)	13 (6.5)	26
		Diploma and above	44 (22.1)	28 (14.1)	72
9	Residence	Urban	124 (62.3)	95 (47.7)	219
		Rural	75 (37.7)	104 (52.8)	179
10	Year of marriage	< 18 years	71 (35.7)	88 (44.2)	159
		≥ 18 years	128 (64.3)	111 (55.8)	249
11	Year of first birth (n = 271)	≤ 19 years	56 (47.9)	87 (56.5)	143
		≥ 20 years	61 (52.1)	67 (43.5)	128

**Table 2:** Obstetric history and utilization of pregnant mothers attending antenatal care follow up in Easter zone of Tigray, 2019

S. no.	Characteristics	Responses	Before or at 16 weeks (n = 199)	After 16 weeks (N = 199)	Total
1	Total no of pregnancy	Only one	82 (41.2)	45 (22.6)	127
		2–4 times	89 (44.7)	105 (52.8)	194
		5 and more	28 (14.1)	49 (24.6)	77
2	Total no of births	Only one	67 (49.6)	58 (35.4)	125
		2–4 births	67 (49.6)	58 (35.4)	125
		5 and more births	15 (11.1)	26 (15.9)	41
3	Total alive births	No alive birth	28 (21.5)	15 (9.1)	43
		1–2 alive births	69 (53.1)	77 (47)	146
		≥ 3 and above alive births	33 (25.4)	72 (43.9)	105
4	Total no of live children	No alive child	28 (21.5)	15 (9.1)	43
		1–2 live children	69 (53.1)	77 (47)	146
		3 and more alive children	33 (25.4)	72 (43.9)	105
5	Ever had still birth	Yes	19 (16.4)	24 (15.6)	43
		No	97 (83.6)	130 (84.4)	227
6	History of abortion	Yes	25 (21.2)	29 (18.8)	54
		No	93 (78.8)	12 (581.2)	218
7	Current pregnancy	Planned	146 (73.4)	114 (57.3)	260
		Unplanned	53 (26.6)	84 (42.2)	137
8	Gravidity	1st	83 (41.9)	46 (23)	129
		2nd	42 (21.2)	47 (23.5)	89
		3rd and above	73 (36.9)	107 (53.5)	180
9	History of ANC follow up for the previous pregnancy	Yes	103 (52)	138 (69)	241
		No	11 (5.6)	16 (8)	27
		Never pregnant	84 (42.4)	46 (23)	130
10	Time of first ANC for previous pregnancy	Before or 16 weeks	53 (50.5)	37 (26.6)	90
		After 16 weeks	35 (33.3)	71 (51.1)	106
		I did not remember	17 (16.2)	31 (22.3)	48
11	No of ANC for previous pregnancy	Only one	9 (8.3%)	7 (5.1)	16
		Two times	15 (13.9)	26 (19.1)	41
		Three times	28 (25.9)	54 (39.7)	82
		Four times	47 (43.5)	36 (26.5)	83
		Above four times	9 (8.3)	13 (9.6)	22
12	Know when ANC will start	Before or 16 weeks	129 (64.8)	44 (22.1)	173
		After 16 weeks	42 (21.1)	116 (58.3)	158
		I did not remember	28 (14.1)	39 (19.6)	67
13	Know advantage of ANC	Yes	185 (92.9)	125 (72.9)	330
		No	34 (17.1)	34 (17.1)	68
14	Know dangers signs of pregnancy	Yes	128 (64.3)	133 (66.8)	261
		No	71 (35.7)	66 (33.2)	137
15	Types of dangers signs (n = 261)	Bleeding	121 (94.2)	109 (80.7)	230
		Sever head ache	53 (41.4)	49 (36.3)	102
		Swelling of extremes	41 (32.2)	38 (28.4)	79
		Convulsion	41 (32.2)	41 (30.6)	82
		Severe abdominal cramping	29 (22.7)	30 (22.4)	59
		Others	55 (42.6)	65 (48.1)	120
16	Accompanied by for the previous ANC follow up	Relatives	32 (30.2)	27 (20.1)	59
		My partner	40 (37.7)	42 (31.3)	82
		WDA	2 (1.9)	10 (7.5)	12
		My self	32 (30.2)	55 (41)	87
17	Partner encourage for ANC visit	Yes	167 (83.9)	152 (76.4)	319
		No	32 (16.1)	47 (23.6)	78
18	Receive inf/n when to start ANC Visit	Yes	144 (72.2)	149 (74.9)	293
		No	55 (27.8)	50 (25.1)	105
19	Experienced danger signs for previous pregnancy	Yes	50 (42.4)	47 (30.7)	97
		No	68 (57.6)	106 (69.3)	174
20	Means of identifying current pregnancy	Amenorrhea	74 (37.2)	84 (42.2)	158
		HCG test	104 (52.3)	94 (47.2)	198
		Told by Health provider	15 (7.5)	16 (8)	31
		Other	6 (3)	5 (2.5)	11
21	Perception of no of ANC visits	Only one	4 (2)	3 (1.5)	7
		Two times	43 (21.6)	48 (24.1)	91
		Three times	98 (49.2)	94 (47.2)	19
		Four times	54 (27.1)	54 (27.1)	108
		More than four times	4 (2)	3 (1.5)	7
22	Time of recognized the current pregnancy	1–2 months	129 (64.8)	55 (27.6)	184
		After 3 months	70 (35.2)	144 (72.4)	211
23	Have HF in your kebelle	Yes	171 (85.9)	181 (91)	362
		No	28 (14.1)	18 (9)	36
24	Distance to the nearest health facility	< 60 min	158 (79.4)	141 (70.9)	299
		60–120 min	25 (12.6)	27 (13.6)	52
		> 120 min	16 (8%)	31 (15.6)	47
25	ANC services is provided in comfortable time	Yes	175 (87.9)	177 (88.9)	352
		No	24 (12)	22 (11%)	46
26	Health provide respectful care	Yes	167 (83.9)	155 (77.9)	322
		No	32 (16.1)	44 (22.11)	77
27	Receive in f/n about Advantage of ANC	Yes	179 (89.9)	178 (89.4)	357
		No	20 (10.1)	21 (10.6)	41
28	Source of information for advantage of ANC	Health providers	141 (77.9)	150 (84.2)	290
		My partner	23 (13.3)	17 (9)	40
		My relatives	24 (13.4)	13 (7.3)	37
		mass media	51 (28.5)	50 (28.1)	101
		Books	16 (8.9)	9 (5.1)	25
		School	12 (8.7)	7 (3)	17
29	In which HF had receive ANC for previous pregnancy	Privet	17 (16.3)	19 (13.9)	36
		NGO	13 (12.5)	17 (12.4)	30

**Table 3:** Determinates of late ANC of pregnant mother attending first visit for ANC at Eastern Zone of Tigray. North Ethiopia, 2019

S. no	Variables	Characteristics	Controls	Cases	COR	AOR	p value
1.	Husband occupation	G. employed	33 (16.6)	31 (15.6)	*1.036 (0.06, 0.912)*	1.4789 (0.334, 6.583)	0.606
		NGO and students	17 (8.5)	9 (4.5)	*1.143 (.024, 0.83)*	4.682 (0.220, 9.405)	0.323
		Farmer	34 (17.1)	41 (20.6)	1.301 (0.079, 1.156)	1.876 (0.491, 7.161)	0.357
		Merchant	51 (25.6)	49 (24.6)	0.94 (0.064, 0.903)	1.203 (0.324, 4.460)	0.783
		Private employed	51 (20.7)	53 (26.5)	1.234 (0.063, 0.87)	1.532 (0.756, 3.425)	0.345
		Daily laborer	13 (6.5)	16 (8.2)	1	1	
2	Residence	urban	124 (62.3)	90 (45.2)	1	1	
		Rural	75 (37.7)	109 (54.8)	*1.984 (1.329, 2.961)*	1.697 (0.368, 1.318)	0.267
3	Current pregnancy status	Planned	145 (72.9)	95 (47.7)	1		
		Unplanned	54 (27.1)	104 (52.3)	*2.940 (1.935, 4.466)*	*4.036 (1.560, 5.671*	*0.001*
5	Time of previous ANC follow up (n = 247)	Before or 16 weeks	65 (61.3)	37 (26.8)	1		
		After 16 weeks	31 (29.2)	71 (51.4)	*4.024 (2.244, 7.214)*	*3.904 (1.982, 7.688*	*0.001*
		I did n’t remember	10 (9.4)	30 (21.7)	*5.270 (2.317, 11.986)*	2.892 (1.165, 7.159)	0.022
6	Partner encourage for ANC visit	Yes	160 (80.4)	127 (63.8)	1		
		No	39 (19.6)	72 (36.2)	*2.294 (1455, 3.614)*	*1.232 (1.051, 4.675)*	*0.047*
7	Experience of danger signs	Yes	52 (44.8)	45 (29)	1		
		No	64 (55.2)	110 (71)	*1.986 (1.200, 2.792)*		
8	HF provided for pr px	Privet	22 (11.1)	26 (13.1)	1.405 (0.187, 0.880)	4.036 (1.381, 11.791)	0.061
		NGO	24 (12.1)	10 (5)	1.060 (0.573, 1.9560)	1.649 (0.189, 2.265)	0.498
		Governmental	153 (76.9)	163 (81.9)	*1*	1	
	Total no of births	Only one	67 (49.3)	58 (35.6)	*1*		
		2–4 births	54 (39.7)	79 (48.5)	*1.690 (1.032, 2.768)*	*1.145 (1.033, 3.708)*	*0.022*
		5 and more births	15 (11)	26 (16)	2.002 (0.969, 4.139)	1.003 (0.296, 3.403)	0.987
9	Time of recognize being pregnant	1–2 months	126 (63.3)	59 (29.6)	1	1	
		≥ 3 months	73 (36.7)	140 (70.4)	*4.166 (2.736, 6344)*	*4.75 (2.495, 9.042)*	*0.001*
10	Distance to nearest HF	< 60 min	159 (79.9)	140 (70.4)	*0.454 (0.239, 0.866)*	1.058 (0.336, 3.392)	0.924
		60–120 min	24 (12.1)	28 (14.1)	0.602 (0.267, 1.358)	0.571 (0.151, 2.152)	0.408
		> 120 min	16 (8)	31 (15.6)	1	1	
11	Adequate time provided for previous pregnancy	Yes	161 (80.9)	150 (75.4)	*0.327 (0. 148, 0.720)*	*0.461 (0.342, 0.875)*	*0.034*
		No	38 (19.1)	49 (24.6)	1	1	

## Abbreviations

AGU: Adigrat University
ANC: ante natal care
AOR: adjusted odds ratio
CI: confidence interval
CMHS: College of Medicine and Health Sciences
SD: standard deviation
SPSS: Statistical Package for Social Sciences
WHO: World Health Organization
